# A *Caenorhabditis elegans* Model for Integrating the Functions of Neuropsychiatric Risk Genes Identifies Components Required for Normal Dendritic Morphology

**DOI:** 10.1534/g3.119.400925

**Published:** 2020-03-04

**Authors:** Cristina Aguirre-Chen, Natalia Stec, Olivia Mendivil Ramos, Nuri Kim, Melissa Kramer, Shane McCarthy, Jesse Gillis, W. Richard McCombie, Christopher M. Hammell

**Affiliations:** Cold Spring Harbor Laboratory, One Bungtown Road, Cold Spring Harbor, NY 11724

**Keywords:** *Caenorhabditis elegans*, autism spectrum disorder, schizophrenia, neuropsychiatric risk genes, RNA interference, dendritic arborization, model organism, neuronal development

## Abstract

Analysis of patient-derived DNA samples has identified hundreds of variants that are likely involved in neuropsychiatric diseases such as autism spectrum disorder (ASD) and schizophrenia (SCZ). While these studies couple behavioral phenotypes to individual genotypes, the number and diversity of candidate genes implicated in these disorders highlights the fact that the mechanistic underpinnings of these disorders are largely unknown. Here, we describe a RNAi-based screening platform that uses *C. elegans* to screen candidate neuropsychiatric risk genes (NRGs) for roles in controlling dendritic arborization. To benchmark this approach, we queried published lists of NRGs whose variants in ASD and SCZ are predicted to result in complete or partial loss of gene function. We found that a significant fraction (>16%) of these candidate NRGs are essential for dendritic development. Furthermore, these gene sets are enriched for dendritic arbor phenotypes (>14 fold) when compared to control RNAi datasets of over 500 human orthologs. The diversity of PVD structural abnormalities observed in these assays suggests that the functions of diverse NRGs (encoding transcription factors, chromatin remodelers, molecular chaperones and cytoskeleton-related proteins) converge to regulate neuronal morphology and that individual NRGs may play distinct roles in dendritic branching. We also demonstrate that the experimental value of this platform by providing additional insights into the molecular frameworks of candidate NRGs. Specifically, we show that ANK2/UNC-44 function is directly integrated with known regulators of dendritic arborization and suggest that altering the dosage of ARID1B/LET-526 expression during development affects neuronal morphology without diminishing aspects of cell fate specification.

Neuropsychiatric disorders are a group of complex and heterogeneous mental diseases that greatly contribute to human morbidity, mortality, and long-term disability (McCarroll *et al.* 2014; Insel and Landis 2013). The genetic architecture of neuropsychiatric disorders, such as schizophrenia, bipolar depression, and autism has been refractive to linkage and candidate-gene association studies to varying degrees (McCarroll *et al.* 2014). However, recent advances in genomic technologies have paved the way for higher-resolution studies that have identified specific genomic regions or candidate genes that may play key roles in the etiology of these syndromes. Significant progress has been made using these methods, which include common-variant association studies (CVAS) (Schizophrenia Working Group of the Psychiatric Genomics Consortium 2014; Ripke *et al.* 2013; Schizophrenia Psychiatric Genome-Wide Association Study (GWAS) Consortium 2011), studies of copy number variation (CNV) (Krumm *et al.* 2012), and exome sequencing (Iossifov *et al.* 2014; Fromer *et al.* 2014; McCarthy *et al.* 2014; Purcell *et al.* 2014). Still, it has been challenging to functionally validate these candidate neuropsychiatric risk genes (NRGs), or model how mutations in these genes impact pathology. This limitation is in striking contrast to other complex diseases, such as cancer or heart disease, where model organism-based studies have played pivotal roles in gene discovery and validation pipelines, and, ultimately, the development of highly effective therapeutics.

While both ASD and SCZ are complex diseases that manifest at distinct behavioral levels, evidence suggests that neuronal morphology is often altered in patients suffering from these diseases (Penzes *et al.* 2011). Disruption of neuronal morphology has inherent implications for both functional changes at individual synapses as well as alteration of circuit level connectivity. ASD has been linked to local hyper-connectivity between neurons with a wholesale decrease in long-range neural connections (Hutsler and Zhang 2010). SCZ is associated with a reduction in both short- and long-range neuronal connectivity (Glantz and Lewis 2000). The basic genes and genetic mechanisms that establish overall neuronal morphology and dendritic arborization are highly conserved in animals. Therefore, it is likely that some of the genes implicated in ASD and SCZ can be modeled in simpler, more genetically tractable organisms. Indeed, many current mouse models of these diseases can recapitulate some of these features (Isshiki *et al.* 2014; Flores *et al.* 2016; Falk *et al.* 2016). Specifically, both NRG knock out models (both in mouse and patient derived-iPSCs) and lesion models (toxin injection) result in specific and similar cyto-architectural abnormalities indicating that structural defects, at both anatomical and cellular levels, impact disease pathology (Bourgeron 2015; Flores-Alcantar *et al.* 2011; Falk *et al.* 2016; Sekar *et al.* 2016; Mei *et al.* 2016). Screening the newly identified NRGs is an important next step in understanding the etiology of neuropsychiatric disease. Given the sheer number of candidate ASD and SCZ genes identified via high-throughput sequencing strategies, dissecting individual candidate NRG function in either mouse models or patient derived-iPSCs remains a daunting task.

Because of its inherent simplicity, the *C. elegans* nervous system is an outstanding model for *in-vivo* functional studies of neuron activity and neuronal development. Its nervous system, which contains 302 neurons, has been fully mapped and is invariant between animals (White *et al.* 1986). Notably, the neurons derived from the posterior V lineage (PVD), sensory neurons that responds to harsh mechanical stimuli (Way and Chalfie 1989) and cold temperature (Chatzigeorgiou *et al.* 2010), exhibit highly elaborate dendritic arborization patterns, forming a web-like pattern over the majority of the animal (Figure 1A)(Aguirre-Chen *et al.* 2011; Albeg *et al.* 2011; Oren-Suissa *et al.* 2010; Smith *et al.* 2010; Tsalik *et al.* 2003; Yassin *et al.* 2001). The stage-specific development of PVD dendrites generates the sequential and orthogonal growth patterns of primary, secondary, tertiary, and quaternary dendrites, giving rise to repeated “menorah” structures (Figures 1A-C). This size and complexity of PVD neurons are ideal for studying the mechanisms that underlie the establishment of cell type-specific dendritic arborization patterns. Forward genetic studies of this model have identified a number of conserved genes and mechanisms that regulate dendritic architecture, suggesting that this platform can be used to identify and integrate additional genes that function in this process (Díaz-Balzac *et al.* 2016; Salzberg *et al.* 2013; Smith *et al.* 2013; Aguirre-Chen *et al.* 2011; Dong *et al.* 2013; Wei *et al.* 2015). Indeed, mRNA sequencing and protein expression data in human and mouse suggest that a number of ASD and SCZ NRGs are highly conserved, are neuronally expressed (Hoischen *et al.* 2014; Krystal and State 2014), and that defective synaptic and/or dendritic morphology has been implicated in neuropsychiatric disorders (Copf 2015, 2016; Kulkarni and Firestein 2012).

**Figure 1:**
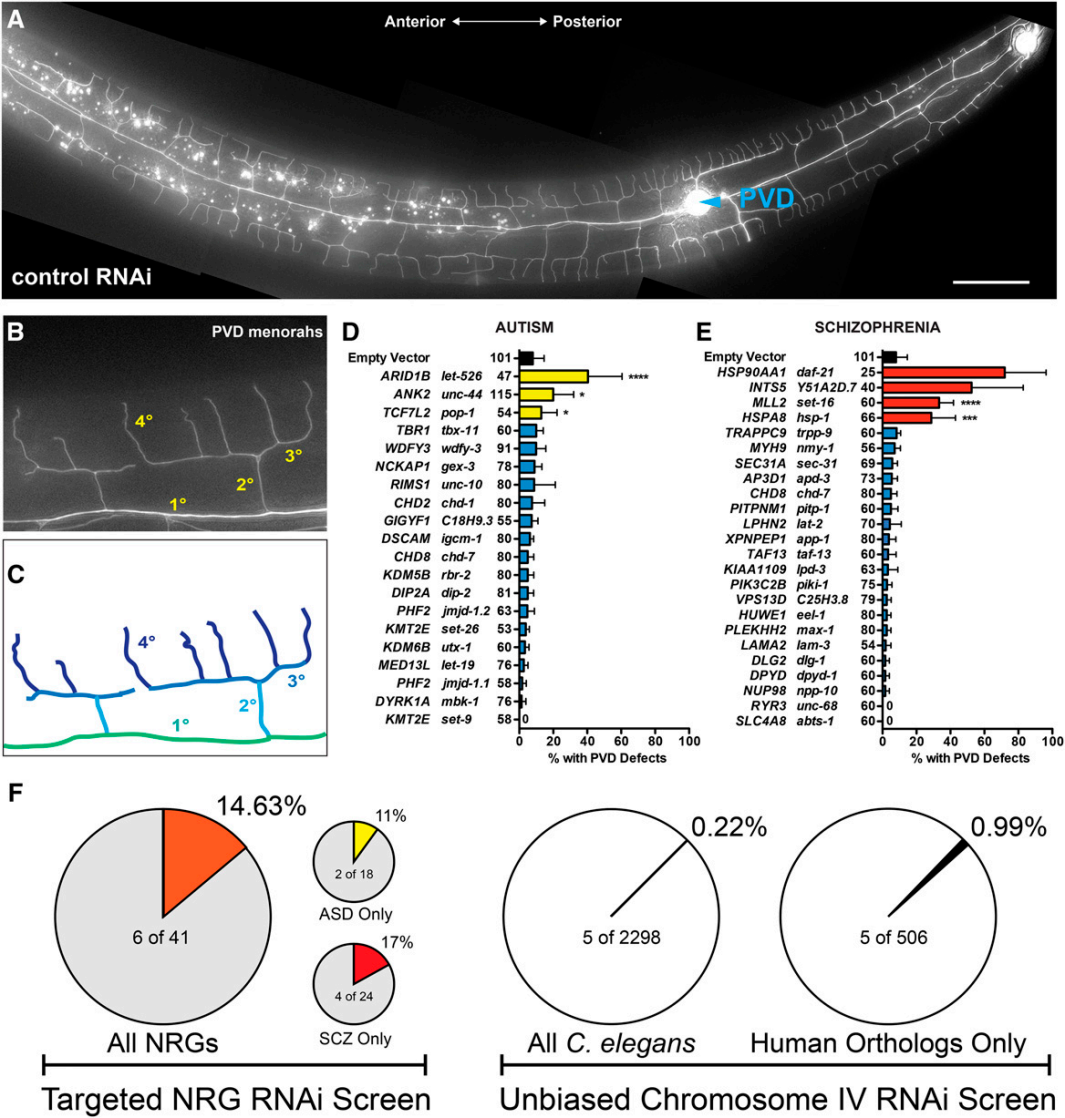
Proper Neuronal Development is Perturbed after the Depletion of *C. elegans* orthologs of ASD or SCZ NRGs. (A) PVD neurons form a web-like dendritic arbor that envelops the body of adult-staged animals. (B,C) PVD menorahs are individual dendritic units that sprout ventrally and dorsally from primary dendrites (1°). Each menorah is composed of secondary (2°), tertiary (3°), and quaternary (4°) dendrites. (D,E) RNAi knockdown of the *C. elegans* orthologs of (D) 3/18 ASD NRGs and (E) 4/24 SCZ NRGs resulted in PVD dendritic arborization or cell-fate specification defects. A single NRG, CHD8, was present in both the ASD- and SCZ-associated lists. Animals exhibiting an overt increase and/or decrease in dendritic branching, a disorganization of the dendritic arbor, or supernumerary PVD cell bodies were scored as defective. For each panel, human NRGs = left column and *C. elegans* orthologs of human NRGs = right column. (F) Comparison of the overall hit rates of ASD and SCZ candidates compared to unbiased screening. Error bars indicate the weighted standard deviation. ****P < 0.0001, ***P < 0.001, *P < 0.05 determined by Fisher’s exact test.

## Materials and Methods

### Orthology assignment

*C. elegans* orthologs of NRGs were identified through the use of the InParanoid (Sonnhammer and Östlund 2015; O’Brien *et al.* 2005) orthology database (v. 8.0). In cases where InParanoid v. 8.0 did not predict a *C. elegans* ortholog for a specific NRG, InParanoid v. 7.0 predictions, via the OrthoList (Shaye and Greenwald 2011) database, were used.

### Strains

*C. elegans* strains were maintained on nematode growth media plates at 20° using standard techniques (Brenner 1974), unless otherwise noted. Genotypes and transgene information for strains used in this study are listed in extended materials and methods. CRISPR editing protocol for *let-526*::*degron* allele construction can be found in extended data.

### RNAi screening

RNAi by feeding was performed as described (Aguirre-Chen *et al.* 2011; Schmitz *et al.* 2007; Hammell and Hannon 2012) with minor modifications. RNAi clones were retrieved from either the Ahringer (Fraser *et al.* 2000) or Vidal (Kamath *et al.* 2003) RNAi library on generated in this study.

### Photodocumentation

Defects in PVD dendritic branching were visualized using a Zeiss Axio Scope.A1 microscope equipped or a Zeiss Axio Observer 7 with a GFP/RFP optical filter sets. Images were captured with Spot Advanced Software, Version 5.2, with an added Extended Depth of Focus module (Axio Scope.A1) with Zen Blue imaging suite.

### Statistics

GraphPad Prism Software, Version 5.0d, was used for all statistical analyses. For each RNAi clone, data from all independent tests were compiled and a weighted percentage (weighted for sample volume) and weighted standard deviation were calculated. RNAi clones were considered positive if the weighted percentage of animals exhibiting PVD arborization defects was statistically different by Fisher’s Exact Test as compared to animals fed the RNAi empty vector, pPD129.36.

### Data availability

The authors will submit all strains generated in this manuscript to the *Caenorhabditis* Genetics Center which is funded by NIH Office of Research Infrastructure Programs (P40 OD010440). All other strains and including bacterial RNAi reagents are available on request. Supplemental material available at figshare: https://doi.org/10.25387/g3.11933190.

## Results

We sought to develop a model to functionally assess the roles of NRGs in developmentally-regulated dendritic arborization by using RNAi against the *C. elegans* orthologs of candidate NRGs in a strain expressing a cytoplasmically localized GFP reporter expressed exclusively in PVD neurons (Figure 1B and C). To benchmark this strategy, we selected predicted candidate NRGs via a literature-based search of ASD (Iossifov *et al.* 2014) and SCZ (Fromer *et al.* 2014; Guipponi *et al.* 2014; Gulsuner *et al.* 2013; McCarthy *et al.* 2014) exome sequencing studies focused on identifying *de novo* variants. We specifically selected candidate NRGs whose mutations were predicted to be largely gene inactivating (Table S1) (Iossifov *et al.* 2014; Fromer *et al.* 2014; Guipponi *et al.* 2014; Gulsuner *et al.* 2013; McCarthy *et al.* 2014) because our RNAi-mediated depletion strategy leads to a partial to strong loss of protein function of target genes. This analysis resulted in 27 ASD and 40 SCZ candidate genes with a single gene shared in both diseases (Figure 1D and E and Tables S2 and S3). Of these 66 non-overlapping ASD- and SCZ-associated NRGs, 41 (62%) were identified as having ≥ 1 *C. elegans* ortholog (Figure 1D and E)(Sonnhammer and Östlund 2015; O’Brien *et al.* 2005; Shaye and Greenwald 2011). In total, 43 *C. elegans* orthologs were depleted via RNAi and, on average, >50 F1 animals per gene were scored for PVD developmental phenotypes. RNAi depletion of 7 (>17%, n = 7/41) candidate NRG orthologs elicited penetrant phenotypes that altered dendritic morphology (6 orthologs with >20% of F1 animals affected) or PVD cell fate specification (1 NRG ortholog) (Figure 1D and 1E and Tables S2 and S3).

We calculated the enrichment of PVD morphological phenotypes in the NRG sets using two approaches. A previous study employed a similar RNAi-based strategy to query the role of 2298 *C. elegans* genes located on chromosome IV (Aguirre-Chen *et al.* 2011) identified 5 genes of 2298 (5/2298, *i.e.*, 0.22% of genes) as well as 6 other candidate genes located at other genomic loci that affect neuronal morphology. As compared the 5 of 2298 genes from chromosome IV that exhibit PVD morphology phenotypes when depleted by RNAi, our hit rate of 14.63% (n = 6/41) represents a >65-fold enrichment over the hit rate from unbiased large-scale RNAi screening (Figure 1F). However, only a subset of the 2298 genes on Chromosome IV have unambiguous human orthologs. Therefore, as a second metric, we calculated the number of one-to-one human orthologs that are present on *C. elegans* chromosome IV. A minimum of 506 direct orthologous gene pairs exist on the chromosome. Using this conservative metric, our NRG set is enriched >14-fold for phenotypes associated with altered neuronal morphology as compared to random human orthologs on chromosome IV (Figure 1F).

### Depletion of NRG orthologs elicits a variety of distinct PVD phenotypes

**Figure 2:**
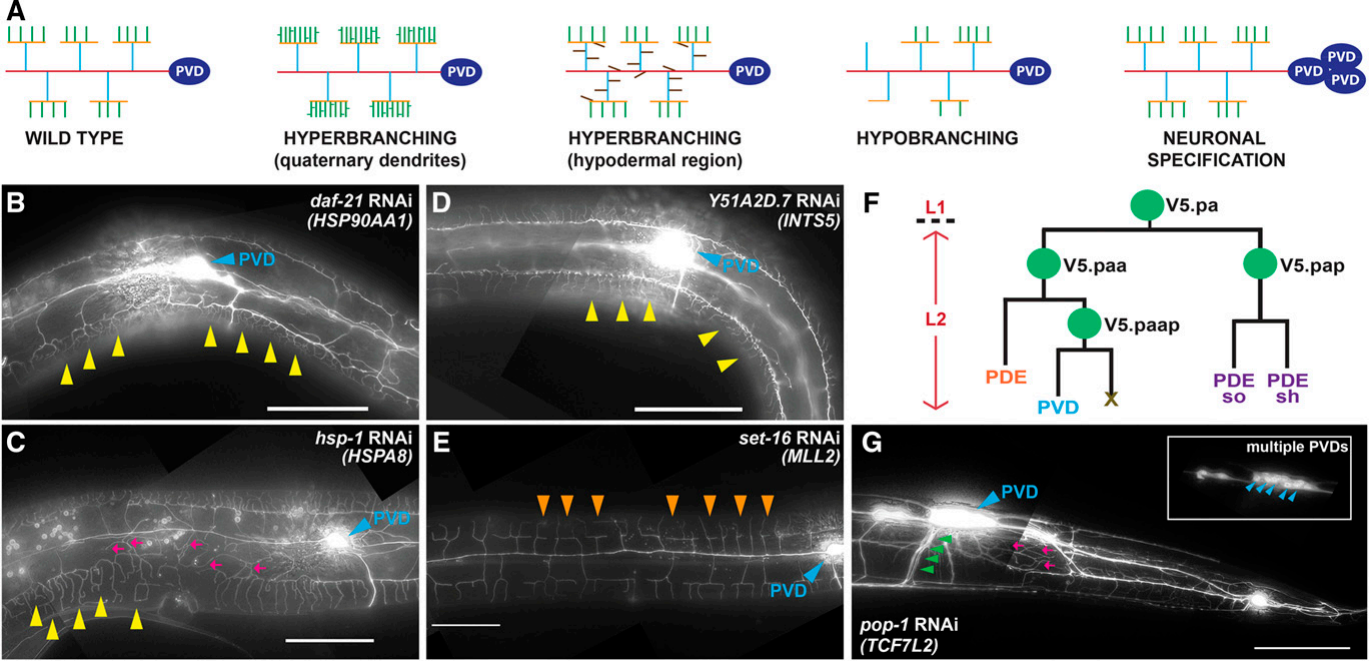
RNAi-mediated Gene Knockdown of NRG Orthologs Disrupts Dendritic Arbor Patterning and Neuronal Cell Fate Specification. (A) Schematic depicting the types of dendritic arborization or cell specification defects scored throughout all candidate-based RNAi screens in this study. RNAi against (B) *daf-21* (*HSP90AA1*) or (D) *Y51A2D.7* (*INTS5*) leads to hyperbranching of 4° dendrites (yellow arrowheads). (C) Animals fed *hsp-1* (*HSPA8*) dsRNA exhibit hyperbranching of 4° dendrites (yellow arrowheads) and increased dendritic branching in the hypodermal region (magenta arrows). (E) RNAi against *set-16* (*MLL2*) results in hypobranching of 4° dendrites (orange arrowheads). (F) Cell lineage diagram depicting stage-specific division patterns that give rise to PVD neurons. (G) *pop-1* (*TCF7L2*) dsRNA-treated animals exhibit supernumerary PVD cell bodies (blue arrowheads, inset) and axons (green arrowheads) along with an increase in dendritic branching (magenta arrows). In panels B, C, D, E, and G, the PVD cell body is labeled with a blue arrowhead and anterior is to the left. Scale bars: 50μm.

PVD architecture is determined by multiple genetic components that converge to regulate the formation of the complex dendritic arbors. Studies in *C. elegans* indicate that mutations in critical genes can lead to an array of phenotypes including loss of dendritic self-avoidance, cell fate alterations, and dendritic hyper- and hypobranching (Figure 2A) (Dong *et al.* 2015). A majority of genes identified through these forward genetic strategies encode structural components (expressed in PVD or cells that directly interact with PVD neurons) that function as membrane-bound, cell surface receptors and/or proteins that mediate interactions between these components and cytoskeletal structures in PVD neurons (Inberg *et al.* 2019). While the NRGs identified in our candidate-based screen also exhibit a wide array of morphological defects, a majority of them encode ubiquitously expressed cellular components (Supplemental Figure S1) that likely function in general cell functions. For instance, loss of three NRG orthologs, *daf-21/*HSP90AA1, *hsp-1**/*HSPA8 (encoding two essential stress-related protein chaperonins) and *Y51A2D.7**/*INTS5 *(*encoding a component of the integrator complex that associates with the C-terminal domain of RNA polymerase II large subunit to ensure proper processing of the splicing-related U1 and U2 snRNAs and mRNA transcription) result in the hyperbranching of secondary, tertiary, and/or quaternary dendrites (Figures 2B, 2C, and 2D). In contrast, depletion of *set-16*/MLL2 (encoding a H3-K4 methyltransferase) results in the reduction of quaternary dendrites (Figure 2E). Interestingly, RNAi depletion of one NRG ortholog leads to complex dendritic arborization phenotypes: RNAi knockdown of *let-526**/*ARID1B (encoding a component of the SWI/SNF chromatin remodeling complex) results in hyperbranching in the dendritic hypodermal region, a lateral region that lies between the dorsal and ventral hypodermal/muscle borders, as well as hypobranching of quaternary dendrites and self-avoidance defects (Table S2).

In addition to dendritic arborization phenotypes, we also identified a single NRG ortholog, *pop-1**/*TCF7L2 (encoding a high mobility group (HMG) box-containing transcription factor that functions in the Wnt signaling pathway), whose RNAi knockdown results in a PVD cell specification defect. In wild-type animals, PVD neurons are derived from the V5 cell lineage through a series of repeated cell divisions that take place at the L2 larval stage (Figure 2F). In *pop-1**/*TCF7L2(RNAi) animals, defective PVD cell specification leads to supernumerary PVD cell bodies and is consistent with other cell fate specification defects of other V cell lineages (Ren and Zhang 2010) (Figure 2G). Animals depleted of *pop-1* also exhibit dendritic hyper-branching and impaired dendritic self-avoidance (Figure 2G). Notably, in contrast to the single ventrally-directed PVD axon seen in wild-type animals, *pop-1**/*TCF7L2(*RNAi*) animals exhibit multiple ventrally-directed axons (Figure 2G), suggesting that a partial overlay of PVD dendritic arbors may account for the dendritic hyper-branching and self-avoidance defects. These findings indicate that *C. elegans* PVD neurons can serve as an effective *in vivo* triage platform for screening orthologs of candidate NRGs, revealing functionally distinct roles in the control of dendritic arbor patterning. Furthermore, this indicates that genes involved in diverse and common cellular functions (transcription, chromatin remodeling, protein folding and cytoskeleton dynamics/transport) converge to regulate normal neuronal morphology.

### Depletion of NRG orthologs in an RNAi hypersensitive background reveals additional genes related to NRGs that disrupt dendritic arborization

**Figure 3:**
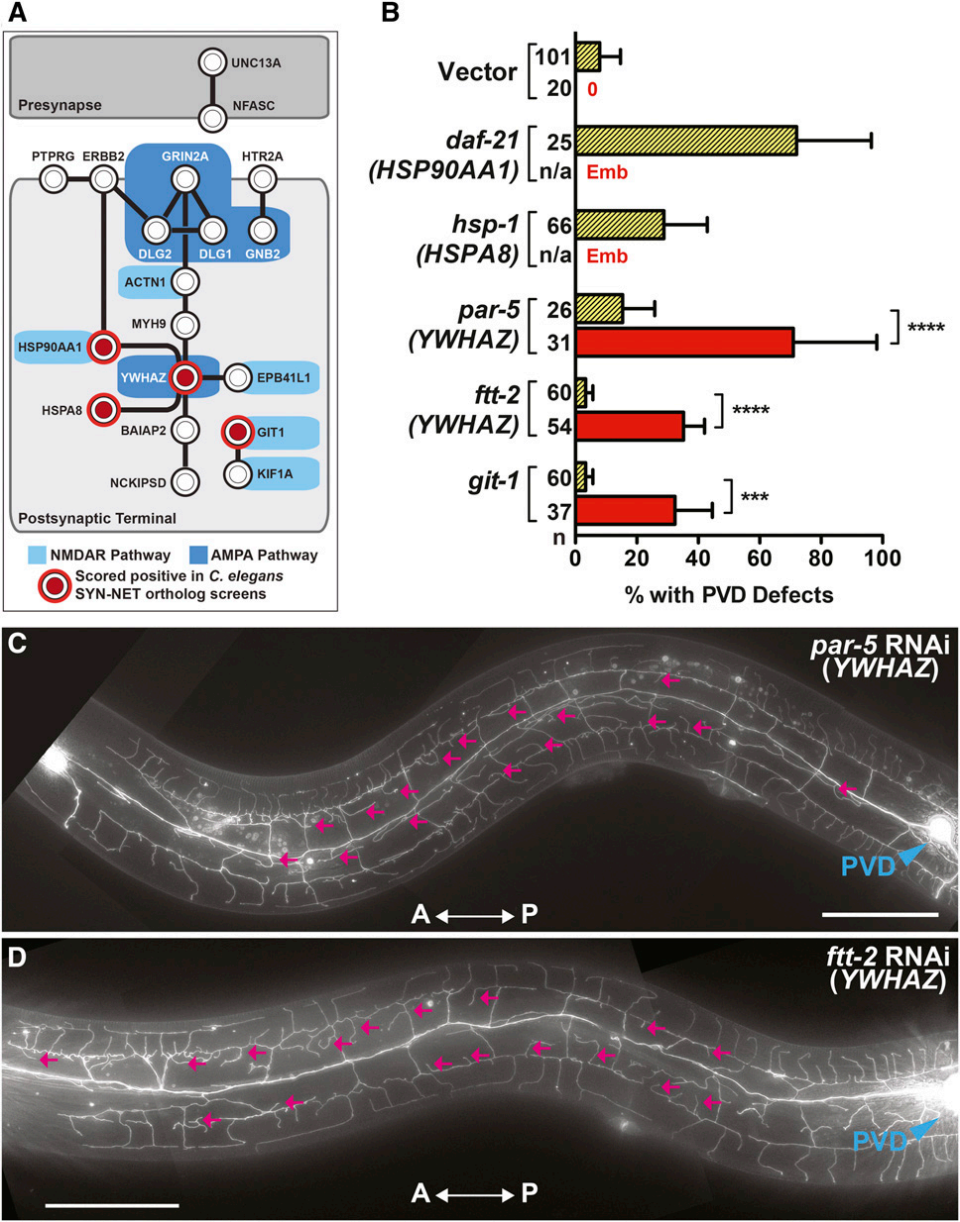
Use of the RNAi Hypersensitive Strain, *nre-1**(**hd20**) **lin-15b**(**hd126**)*, offers a Complementary Approach to Identifying Additional NRG Orthologs that Regulate Dendritic Development. (A) A synaptic protein-protein interaction network in which SYN-NET NRG interactions were identified via the SynSysNet Database (http://bioinformatics.charite.de/synsysnet/). Circles demarcated in red are positive hits identified through the use of the wild-type or the *nre-1**(**hd20**) **lin-15b**(**hd126**)* RNAi hypersensitive strain. (B) RNAi of all SYN-NET NRG orthologs identified four NRGs (represented by five *C. elegans* orthologs) as novel regulators of dendritic branching in either the wild-type (yellow bar with diagonal lines) or *nre-1**(**hd20**) **lin-15b**(**hd126**)* RNAi hypersensitive (red bar) background. (C and D) *YWHAZ* orthologs, *par-5* and *ftt-2*, exhibit increased dendritic branching in the hypodermal region (magenta arrows). Anteroposterior orientation is indicated by the white double-headed arrow. In panels d and e, the PVD cell body is labeled with a blue arrowhead. n, number of animals scored. Error bars indicate the weighted standard deviation. ****P < 0.0001, ***P < 0.001 determined by Fisher’s exact test. Scale bars: 50μm.

Previous work has shown that RNAi knockdown of neuronally-expressed genes inefficient in *C. elegans*, suggesting that RNAi in wild-type genetic backgrounds may underestimate the roles of particular genes in neuronal function and development (Schmitz *et al.* 2007; Wang *et al.* 2005). Therefore, we complemented our RNAi experiments in wild-type animals with those done in a well-characterized RNAi hypersensitive genetic background (*nre-1**(**hd20**) lin15b(**hd126**))* to address three additional questions. First, we determined if using the enhanced efficiency of RNAi in these strains could increase the penetrance or expressivity of the PVD dendritic morphology phenotypes of candidate NRGs. Indeed, depletion of *unc-44* (*ANK2)* in RNAi hypersensitive animals results in an increase in both the penetrance and expressivity of PVD RNAi phenotypes as compared to wild-type animals (Aguirre-Chen *et al.* 2011)(Supplemental Figure S2). In addition, we find that depletion of two candidate NRG orthologs, *daf-21*/HSP90AA1 and *hsp-1*/HSPA8, leads to a more severe embryonic lethal (Emb) phenotype in the enhanced RNAi background, rather than altering dendritic phenotypes (Figure 3A and Table S4). The more severe developmental phenotype demonstrates the utility of using both wild-type and RNAi hypersensitive strains for NRG ortholog screening. Importantly, while the enhanced RNAi strain can reveal additional phenotypes associated with NRGs, it does not capture morphological phenotypes of all genetic mutants that have been implicated in dendritic guidance (*e.g.*, *gex-3*) (Zou *et al.* 2018).

Protein-protein interaction networks and additional systems-level/computational organization of NRGs have led to a variety of models for how the disruption of NRG networks may alter neuronal function. For instance, the NMDAR (N-methyl-D-aspartate (NMDA) receptor) and ARC (Activity-regulated cytoskeleton-associated protein) complexes (assembled through boot-strapping two-hybrid, protein-protein interaction assays) have been implicated in SCZ with the assumption that mutations in these genes would alter specific protein-protein associations involved in synaptic communication (Figure 3C)(Glessner *et al.* 2010; Fromer *et al.* 2014; Malhotra *et al.* 2011). While PVD neurons are not thought to be post-synaptic to other neurons and not predicted to express NMDA receptors (Brockie *et al.* 2001)(Albeg *et al.* 2011), we employed the RNAi hypersensitive strain to functionally probe additional orthologous genes implicated in NMDAR and ARC networks (Figure 3B) for roles in controlling dendritic arborization. Through this approach, we found that the orthologs of two additional neuro-psychiatric-associated risk genes, *YWHAZ* (a 14-3-3 family protein implicated in cytoplasmic signaling) and *GIT1* (encoding a GTPase-activating protein from the ADP ribosylation factor family and likely functions as a scaffold in cytoplasmic vesicle trafficking), are required for proper PVD dendritic arborization (Figure 3A, C and D). *YWHAZ* and *GIT1* are represented by three *C. elegans* orthologs, *par-5*, *ftt-2*, *and **git-1*, respectively. RNAi knockdown of each results in hyper-branching in the hypodermal region (Figure 3A). These phenotypes were substantially less penetrant when depleted in the wild-type RNAi background (Figure 3A) and suggest that the ubiquitously expressed genes (Cao *et al.* 2017) encoding downstream components of the NMDAR and ARC complexes also contribute to the production of normal neuron morphology.

Third, we used the hypersensitive strain to determine if knocking down candidate NRGs altered other aspects of neuronal development in addition to dendritic structures. For NRG candidates where RNAi does not lead to embryonic lethality (*unc-44*, *ftt-2*, and *git-1*), we monitored the architecture of DA/DB cholinergic motor neurons that born embryonically and by the end of the L1 stage, extend axon commissures circumferentially from the ventral nerve chord to the dorsal nerve chord (Supplemental Figure S3A). RNAi treatment failed to reveal any significant DA/DB architectural phenotypes using RNAi conditions identical to those that elicit severe PVD branching phenotypes in F1 animals (Supplemental Figure S3B). These results are generally consistent with previous studies indicating that most genes required for normal axon migration do not play a significant role in PVD arborization (Schmitz *et al.* 2007; Aguirre-Chen *et al.* 2011). To determine if additional NRGs may also play roles axon guidance, we depleted candidate NRGs using post-embryonic RNAi feeding and monitored the ventral axon extension phenotypes of HSN neurons. During the late L4 stage, HSNs extend a single axon projection toward the ventral nerve chord (VNC). During this process, the HSN neuron forms synapses with vulval muscles and the VC4 and VC5 motor neurons before further extending to the far anterior nerve ring (White *et al.* 1986). When we examined the HSN axon extension phenotypes in animals subjected to post-embryonic RNAi of the ten NRGs that disrupt PVD morphology. Only three of the seven assayable genes (*pop-1**/*TCF, *let-526*, and *unc-44*) exhibited detectable RNAi-induced abnormalities in HSN axon migration (Supplemental Figure S3C and D). Depletion of *hsp-1**/*HSP8A, *daf-21/*HSP90AA1 *and **set-16**/*MML2 in this genetic background lead to larval lethality which precluded the scoring of HSN axon extension phenotypes. While these experiments indicate that three of the genes we identify as playing a role in the control of dendritic arborization also function in axon migration, the HSN migration phenotypes in *pop-1**/*TCF7L2 and *let-526**/*ARID1B RNAi animals are likely due to cell fate defects in vulval precursor cells (animals displaying the HSN phenotype exhibited cell fate specification of vulval precursor or lacked vulval structures completely). It has been previously established that these cell types provide morphogenic cues for growing axons (Garriga *et al.* 1993).

### Genetic mutants of NRG orthologs phenocopy the RNAi-induced dendritic arborization defects

**Figure 4:**
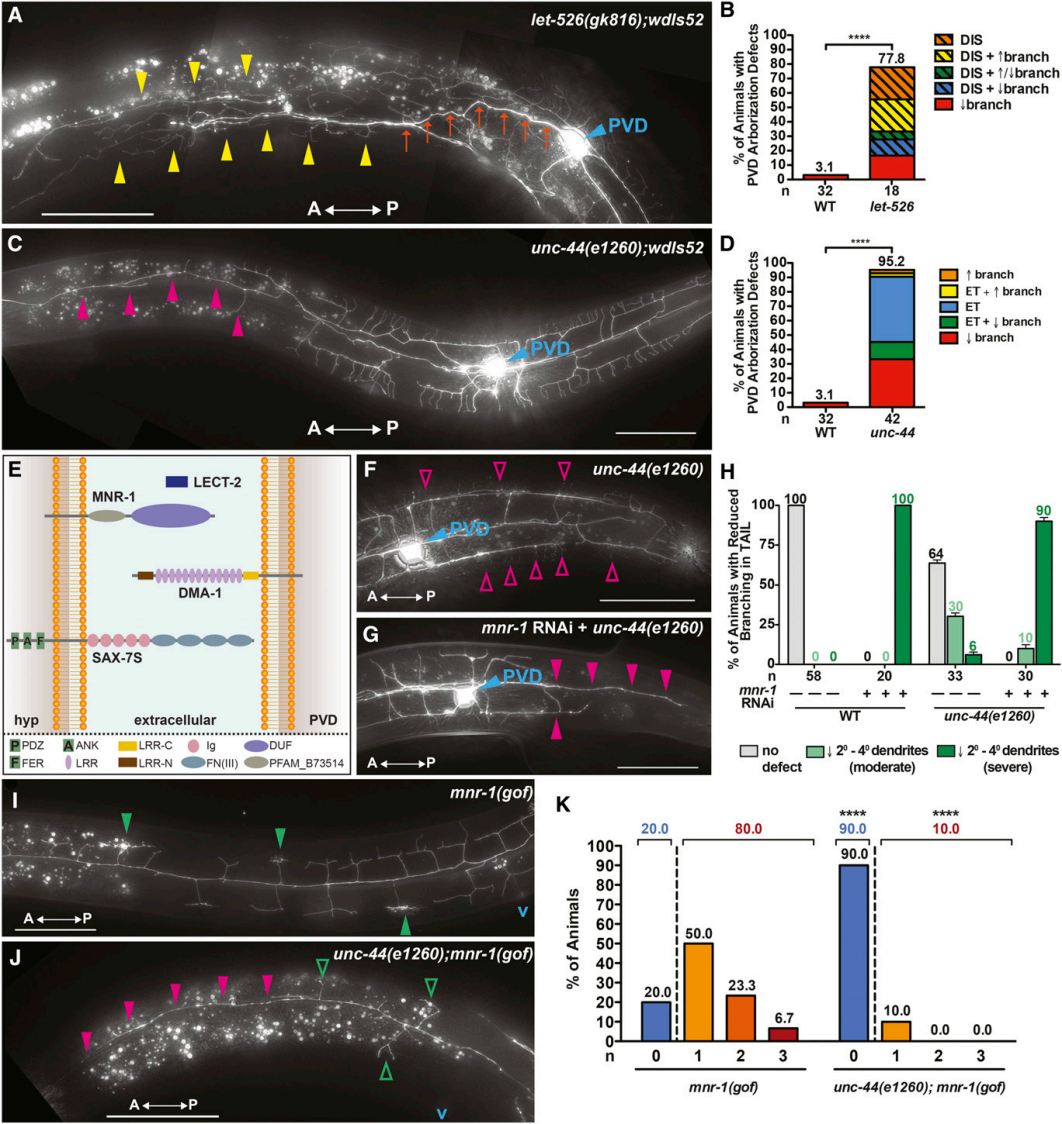
Genetic Mutants Phenocopy RNAi-induced Dendritic Arborization Defects and Genetic Interaction Studies Indicate that *unc-44* (*ANK2*) is Required for SAX-7/MNR-1/LECT-2/DMA-1 Signaling. (A,B) *let-526**(**gk816**)* genetic mutants primarily exhibit a marked disorganization of the dendritic arbor, including spurious branching (yellow arrowheads) and misguidance of the 1° dendrite (orange arrows). A general increase and/or decrease in dendritic branching may also accompany the disorganization phenotype. (C,D) In addition to early termination of the 1° anterior dendrite (not shown), *unc-44**(**e1260**)* genetic mutants may also exhibit a loss of 2°, 3°, and 4° dendrites (magenta arrowheads) at the anterior end of the arbor. (E) Schematic depicting the SAX-7S/MNR-1/LECT-2/DMA-1 multi-protein signaling complex along with protein domains. (F) *unc-44**(**e1260**)* genetic mutants largely retain the ability to form 4° dendrites (magenta open arrowheads) in the tail region, although 6% exhibit a complete reduction in 2°, 3°, and 4° dendrites (H). (G,H) A complete reduction in 2°, 3°, and 4° dendrites (magenta arrowheads) in the tail region is exhibited by 90% of *unc-44**(**e1260**)* mutants treated with *mnr-1* dsRNA. (I,-K) 80% of *mnr-1**(gof)* animals elaborate ≥ 1 baobab (green arrowheads) anterior to the vulva, while 10% of *unc-44**(**e1260**);**mnr-1**(gof)* animals sprout ≥ 1 baobab (green open arrowheads) in this region. *mnr-1**(gof)* animals harboring the *unc-44**(**e1260**)* allele also exhibit a loss of 2°, 3°, and 4° dendrites (magenta arrowheads) at the anterior end of the PVD arbor. Anteroposterior orientation is indicated by the white, double-headed arrow. In panels A, C, F, and G, the PVD cell body is labeled with a blue arrowhead, and “v” in panels i and j marks the approximate location of the vulva. In panels b, d, and h, “n” is the number of animals scored. In panel K, “n” is the number of baobabs present in each genetic background. ****P < 0.0001 determined by Fisher’s exact test. Scale bars: 50μm.

To validate dendritic patterning phenotypes associated with the depletion of candidate NRG orthologs, we analyzed available genetic mutant alleles of *let-526* and *unc-44*, the *C. elegans* orthologs of *ARID1B* and *ANK2*, respectively. *C. elegans **let-526* encodes an ortholog of human *ARID1B*, an ARID (AT-Rich Interacting Domain) domain-containing subunit of the ATP-dependent BAF-B (BRG1/BRM-associated f actors; mammalian SWI/SNF) chromatin remodeling complex (Wang *et al.* 2004; Nie *et al.* 2003). The putative *let-526* null allele, *let-526*(*gk816*), harbors a 1268bp deletion/5bp insertion that ablates the conserved BRIGHT, ARID (A/T-rich interaction domain) DNA-binding domain. As with *let-526* RNAi (Table S2), maternally rescued *let-526**(**gk816**)* animals segregating from heterozygous parents exhibit a variety of PVD architectural defects, including hyperbranching, hypobranching, and dendritic arbor disorganization (Figure 4A and 4B).

In addition to validating the RNAi phenotype of the global transcription regulator *let-526**/*ARID1B, we also sought to validate RNAi phenotypes associated with orthologs of candidate NRGs that encode cytoskeletal proteins that are likely involved in intracellular organization and receptor transport that is critical for cell-cell communication. The *C. elegans* ortholog of *ANK2*, *unc-44*, encodes a set of ankyrin-like proteins (Otsuka *et al.* 1995) that play key roles in axon outgrowth and guidance (Hedgecock *et al.* 1983; Siddiqui and Culotti 2007; Zallen *et al.* 1999), neuronal positioning (Zhou *et al.* 2008), and axon/dendrite trafficking (Maniar *et al.* 2011). Consistent with our previous *unc-44* RNAi findings, we find that animals harboring a strong loss-of-function (premature truncation) allele of *unc-44*, *unc-44**(**e1260**)*, exhibit PVD dendritic hypobranching (Figure 4C and 4D). Almost half of *unc-44**(**e1260**)* animals exhibit an early termination of the dendritic arbor (Figure 4C and D). The penetrance and severity of the hypobranching phenotype is also increased in these genetic mutant animals when compared to those elicited by RNAi (Supplemental Figure S2). This included a dramatic decrease in PVD dendrite branching anterior to the PVD cell body and a complete loss of secondary, tertiary, and quaternary dendrites in the distal region (anterior to the vulva) of the PVD arbor (Figure 4C and D). We also find that the genetic mutants exhibit both dendritic hypobranching distal to the PVD cell body and early termination of the primary anterior and/or posterior dendrite (Figure 4D).

### Genetic interaction studies reveal that unc-44(ANK2) is required for SAX-7/MNR-1/DMA-1 signaling

Genetic studies have demonstrated that proper PVD dendritic arborization is dependent on a quad-partite complex of proteins found at the PVD dendrite/epidermal interface along the length of the animal. DMA-1, encoding a conserved leucin-rich repeat extracellular domain receptor that is expressed on the surface of developing PVD dendrites and binds three additional proteins. Two of these components, the immunoglobulin superfamily cell adhesion molecule SAX-7/LCAM1 and a conserved transmembrane protein, MNR-1 expressed on a localized surface of hypodermal cells and a third, secreted component, LECT-2, is expressed from adjacent muscle cells (Díaz-Balzac *et al.* 2016; Salzberg *et al.* 2013; Dong *et al.* 2013; Liu and Shen 2011) (Figure 4E). Notably, animals harboring a null allele of any component of this signaling complex exhibit a severe PVD dendritic hypobranching phenotype (Díaz-Balzac *et al.* 2016; Salzberg *et al.* 2013; Dong *et al.* 2013; Liu and Shen 2011; Salzberg *et al.* 2013). *unc-44* genetic mutant animals similarly exhibit a reduced-branching phenotype (Figure 4C) and previous reports in indicate that UNC-44 and SAX-7 physically interact (Zhou *et al.* 2008). Therefore, we attempted to probe whether *unc-44* acts cooperatively with the SAX-7/MNR-1/LECT-2/DMA-1 complex by depleting *mnr-1* in an *unc-44**(**e1260**)* genetic mutant background. In contrast to 6% of *unc-44**(**e1260**)* mutants treated with the L4440 empty RNAi vector (n = 2/33; Figure 4F-H), 90% of *mnr-1**(RNAi);**unc-44**(**e1260**)* animals exhibit a severe reduction in higher-order (secondary, tertiary, quaternary) branching in the posterior PVD arbor (tail region) (n = 27/30; Figure 4G, and 4H). In addition, the percentage of *mnr-1**(RNAi);**unc-44**(**e1260**)* animals exhibiting a moderate decrease in higher-order branching is markedly reduced as compared to *unc-44**(**e1260**)* mutants fed L4440 (10%, n = 3/30 *vs.* 30%, n = 10/33) (Figure 4H), further confirming a shift in the severity of the phenotype. Because 100% of wild-type animals treated with *mnr-1* dsRNA exhibit a severe decrease in dendritic branching (n = 20; Figure 4H) that is identical to that observed in *mnr-1**(RNAi);**unc-44**(**e1260**)* animals, these data suggest that *mnr-1* is epistatic to *unc-44* and suggest that *mnr-1* and *unc-44* genetically interact to regulate PVD dendritic branch formation.

In order to further test whether *unc-44* is required for SAX-7/MNR-1/LECT-2/DMA-1 signaling, we exploited a transgenic strain in which the MNR-1 receptor is ectopically expressed in muscle cells (Díaz-Balzac *et al.* 2016). Expression of MNR-1 in this tissue leads to the formation of ectopic “baobabs”, defective PVD menorahs characterized by highly-disorganized and tangled quaternary dendrites that are directly apposed to the MNR-1-expressing muscle cells. Consistent with an essential role for *unc-44* in establishing both normal arbors and ectopic “baobabs,” we find that *unc-44**(**e1260*);*mnr-1**(gof)* animals exhibit a marked reduction of both wild-type menorahs and baobab structures anterior to the vulva (Figure 4I-K). Quantification of this phenotype reveals that 80% of *mnr-1**(gof)* animals elaborate ≥ 1 baobab anterior to the vulva, while 20% are devoid of baobab structures (n = 24/30 and n = 6/30, respectively; Figures 4I and 4K). In contrast, 10% of *unc-44**(**e1260**);**mnr-1**(gof)* animals elaborate ≥ 1 baobab anterior to the vulva, while a complete absence of baobabs is observed in 90% of animals (n = 2/20 and n = 18/20, respectively; Figures 4J and 4K). All together, these genetic interaction studies underscore the key roles that cytoskeleton-associated proteins like UNC-44, in conjunction with the *sax-7**/**mnr-1**/**lect-2**/**dma-1* multi-protein signaling complex, play in PVD dendritic arbor development.

### Normal dendritic architecture is sensitive to changes in LET-526(ARID1B) gene dosage during development

ASD and SCZ risk genes are unique among other genetic etiologies: the causative, loss-of-function mutations typically affect only one of the two copies of the gene (Iossifov *et al.* 2014; Fromer *et al.* 2014; Guipponi *et al.* 2014; Gulsuner *et al.* 2013). Furthermore, a large fraction of the ASD- and SCZ-associated variants occur in what are generally considered essential genes (Blake *et al.* 2011). There are multiple possible mechanisms for how these behavioral and/or neuronal phenotypes could arise. For example, allele-specific gene expression in neurons could generate mosaic animals composed of cells that either express or do not express the wild-type, functional version of the gene. ASD or SCZ phenotypes would, in this case, emerge at a systems level as the integration of defective neurons within an otherwise normal circuit with altered behavioral outcomes. Alternatively, these complex ASD and SCZ behavioral phenotypes may simply result from the cell type-specific haploinsufficiency of specific risk genes. In this scenario, one functional version of an NRG would be sufficient for most somatic cell types to be phenotypically wild-type, but more complex cell types (*e.g.*, neurons) may exhibit a more profound disruption in function at reduced gene dosage.

To address this question using our *C. elegans* PVD model, we took advantage of an experimental system where expression levels of virtually any target protein can be modulated at the post-translational level by an exogenously added ligand. The *A*uxin *I*nducible *D*egradation (AID) system is a novel three-component system in which the proteins harboring a small (44aa) epitope (added via CRISPR/Cas9 genomic editing) can be targeted for degradation by ubiquitin-mediated protein degradation. The specificity of this system is provided by a mRuby-tagged, heterologously-expressed E3-ubiquitin conjugating enzyme from *A. thaliana*, *TIR1*::*mRuby*. Interactions between TIR1 and its targets are regulated in an allosteric manner through the binding of auxin to TIR1 (Dharmasiri *et al.* 2005). This strategy enables the titration of target gene dosage during *C. elegans* development as the specific turnover of the target protein in auxin conditions is both rapid and dosage sensitive across all *C. elegans* tissues including neurons (Zhang *et al.* 2015)(Bhattacharya *et al.* 2019).

**Figure 5:**
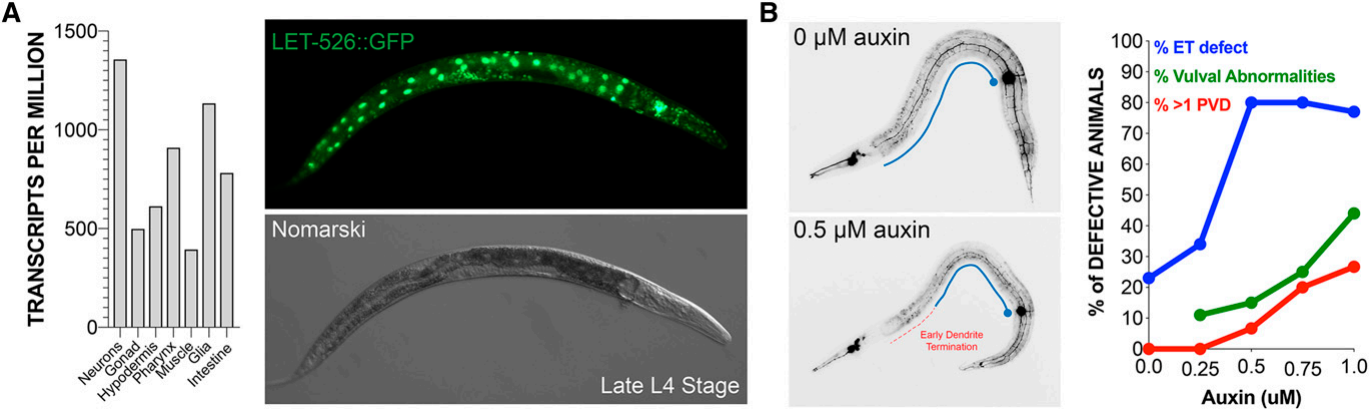
Dendritic architecture is extremely sensitive to reductions in LET-526/ARID1B expression. (A) Single cell RNA-seq experiments (Cao *et al.* 2017) indicate that *let-526* mRNAs are highly expressed in all major tissue types in developing animals. Consistent these results, LET-526::GFP is also broadly expressed. (B) Animals were treated with a gradation of auxin during development. Relatively low concentrations of auxin exposure to animals harboring a *let-526*::*degron* allele resulted in dendritic arborization phenotypes that mirrored those found in *let-536(lf)* mutant animals. The neuronal morphology defects during PVD development were elicited at much lower concentrations of auxin that are needed to observe other pleiotropic somatic phenotypes. ET= extension defects.

We chose to evaluate the AID system in our PVD assay by modulating the expression of broadly-expressed chromatin modifier, LET-526, during larval development (Figure 5a) (Uhlén *et al.* 2015; Cao *et al.* 2017; Celen *et al.* 2017). Mice that are heterozygous for ARID1B display a number of ASD-like behavioral phenotypes, and haploinsufficiency of ARID1B alters neuronal transcription during development (Shibutani *et al.* 2017). In addition, shRNA-mediated knockdown of ARID1B *in vivo* suppresses dendritic arborization in cortical and hippocampal pyramidal neurons similar to its effect on PVD neurons in developing *C. elegans* larvae (Ka *et al.* 2016). To specifically target LET-526, we used the rapid CRISPR-mediated editing and homologous repair method to add the 44aa degron tag to the final exon of the endogenous *let-526* gene (Dickinson *et al.* 2013). The tag does not disrupt gross function of *let-526*: animals that are homozygous for the fusion are superficially wild-type for development and are fertile. We validated that *let-526* is an essential gene by hatching *let-526*::*degron* animals onto growth media that contains saturating concentrations of auxin (1mM). In these conditions, 100% of *let-526*::*degron* animals rapidly matured to adults within 55 hr in no auxin media, whereas 100% of *let-526*::*degron* animals initiate development in a normal fashion but rapidly arrest and die within 24-30 hr on auxin containing growth medium (1mM).

To determine how modulating the dosage of LET-526 altered aspects of development, we employed lower concentrations of auxin in the growth medium (0µM – 1µM) and scored a variety of phenotypes including developmental pace, vulval cell fate specification, and dendritic architecture. At all conditions assayed, animals were viable and developed to adulthood. At 1µM auxin concentrations, developmental pace was slower compared to the no auxin control animals or wild-type animals grown on auxin media. At 53.5 hr after initiation, only 60% of auxin-treated *let-526*::*degron* animals had reached adulthood in auxin conditions, while 100% of non-auxin treated *let-526*::*degron* animals were fertile adults. At low concentrations of auxin (0.25 to 0.50 µm), a minor fraction of animals (∼15%) exhibit defects in vulva morphology and 2 of 30 animals (∼7%) exhibited an ectopic PVD neuron (Figure 5). These phenotypes increased slightly when animals were exposed to higher auxin concentrations. In contrast to these mild developmental defects, PVD morphology was highly sensitive to even low amounts of auxin in the growth medium. At concentrations of auxin as low as 0.5µM, 80% of PVD neurons in young adult animals failed to extend anteriorly (Figure 5B). Elevating auxin concentrations in growth medium did not increase the penetrance of the early termination phenotypes suggesting that dendritic extension is fully compromised at lower concentrations. The early termination defects were also accompanied by other neuronal architectural defects including hyperbranching of arbors in the hypodermal region as well as a lack of self-avoidance of terminal dendrites. These secondary phenotypes increased when animals were raised in high concentrations of auxin (0.75µM to 1µM). This suggests that, like haploinsufficiency in mouse cell models, the establishment of normal dendritic morphology may be highly sensitive to a LET-526/ARID1B expression levels and that a reduction of LET-526 expression during larval development can have differential effects in distinct tissues, specifically impairing neuronal development and architecture while leaving the development of most other somatic cells intact.

## Discussion

Genome and exome sequencing are currently the first step in identifying genetic variants associated with ASD and SCZ. However, the utility of this genomics-level data are limited by the lack of available *in vivo* models to integrate the function of NRGs into molecular and/or systems-level frameworks. In this manuscript, we outline a simple system that can be used to triage NRGs and assign roles in controlling neuronal morphology. This PVD neuron assay is a highly suitable, first pass approach for probing NRG function *in vivo*. This experimental platform is rapid, enabling high content screening of tens to hundreds of candidate genes using an inexpensive technology (RNAi through bacterial ingestion) and minimal experimental equipment (compound microscope) in days to weeks. It provides a platform for determining the importance of a particular NRG in neuronal development, while providing an opportunity to assess a range of highly classifiable neuron morphological phenotypes (Figure 2). This diversity of architectural defects suggests that many candidate NRGs (and their proposed molecular functions in transcription, chromatin remodeling, protein folding, and cytoskeletal activity) may act at distinct phases or processes during normal dendrite development. Importantly, the model is also quantitative, providing the ability to gauge differences in the penetrance and expressivity of structural defects.

A major feature of the PVD dendritic arbor model is that the genetic tractability of *C. elegans* readily enables the integration of individual NRGs with other genes that function during normal neurogenesis. Specifically, we demonstrated that *unc-44* (ANK2) functions with the conserved receptor complex (SAX-7/MNR-1/LECT-2/DMA-1) that mediates interactions between multiple cell types (neuronal and hypodermal) to control dendritic branching (Figure 4). Interactions between distinct cell types are difficult to recapitulate using *in vitro* models of isolated, cultured neurons. This limitation is absent from our *in vivo* system. This is an especially important connection as mutations and deletions in L1CAM, a structural ortholog of SAX-7, have already been implicated in dendritic arborization (Patzke *et al.* 2016), mutations in L1CAM-associated pathway components have already been implicated in autism spectrum disorder (An *et al.* 2014) and the *C. elegans* orthologs of ANK2 and L1CAM (*unc-44* and *sax-7*, respectively) directly and functionally interact in other aspects of neuronal development (Zhou *et al.* 2008). Although not addressed in this manuscript, the inherent genetic tractability of our system will also enable genetic studies between NRGs though co-depletion of target genes or combining engineered genetic mutants with RNAi. This system can also directly complement purely computational approaches aimed at organizing NRGs into functional classes. Through our characterization of the NMDAR (N-methyl-D-aspartate (NMDA) receptor) and ARC (Activity-regulated cytoskeleton-associated protein) complexes, we have demonstrated that, in addition to their potential roles in controlling post-synaptic activity of specific neurons, several of the genes within these pathways are also required for normal dendritic branching. Understanding how distinct NRGs function together through epistasis experiments and tests of genetic redundancy and synergy within these pathways will greatly improve our understanding of the complex genetic landscape in ASD and SCZ.

One intriguing finding from this study is the observation that a vast majority of NRGs that exhibit neuronal morphology phenotypes in our assay are essential for normal development and ubiquitously expressed in both *C. elegans* and mammals (Supplemental Figure S1) (Blake *et al.* 2011). This is especially interesting given that each *de novo* NRG candidate tested in our assay harbor a lesion in only a single copy of the gene in patient samples (Iossifov *et al.* 2014) (Fromer *et al.* 2014; Guipponi *et al.* 2014; Gulsuner *et al.* 2013; McCarthy *et al.* 2014), suggesting haploinsufficiency of individual NRGs results in ASD and SCZ phenotypes. Importantly, eliciting partial loss-of-function phenotypes, a common feature of RNAi experiments, has been instrumental in characterizing aspects of gene function. For example, modulating gene dosage of essential cytoskeletal components via RNAi reveals a range of specific cellular phenotypes during early development whose penetrance and expressivity correlate with the level of actin depletion (Velarde *et al.* 2007). Similarly, the distinct neuronal defects elicited by depletion of individual NRGs via RNAi may indicate that neurons are particularly sensitive to alterations in the expression levels of genes that function in normal, essential facets of cellular physiology. Given the unique functions of neurons, they may place higher demands on diverse cellular systems, including the control of morphological development on physical scales that exceed those of virtually any other cell type (centimeters to meters as compared to micrometers for other somatic cells) and the integration of these features at greater than 1000 sites per cell. Therefore, aspects of transcription (*Y51A2D.7*/INTS5, *ftt-2*/YWHAZ, *par-5*/YWHAZ, and *pop-1*/TCF7L2), chromatin modification (*set-16*/MLL2, *let-526*/ARID1B), protein folding (*hsp-1*/HSPA8 and *daf-21*/HSP90AA1), and cytoskeletal structure (*git-1*/GIT1 and *unc-44*/ANK2) may need to be optimal to establish the architectures of highly-branched dendritic arbors and axon extensions during development. Further work will be necessary to fully characterize the significance of the fine-grained phenotypes observed using RNAi due to the possibility that some are the result and are dependent on partial knock-downs of the targeted gene.

Finally, we also describe how our system can directly address some of the more complex genetic features of NRG function through the ability to directly control the expression of candidate NRGs during *C. elegans* development. Specifically, we employed a novel, ligand-dependent degradation system to query how alterations in gene dosage compromise normal neuronal development (Figure 5). While the AID system would be an impractical alternative to RNAi for primary screening (due to the necessity to genetically engineer each candidate gene with the AID-tag), it will likely prove to be a powerful approach to complement RNAi for further characterization. This system will likely be especially important to define the cell type(s) in which a particular NRG functions as *in vivo* dendritic arborization is modulated by other cell-cell interactions. This would be accomplished by expressing the TIR1 E3 ligase from cell type-specific promoters (Zhang *et al.* 2015). An important feature of the TIR1/AID system is that it leads to the rapid destruction of its target genes. Therefore, this system can also be used to determine the temporal requirements of an NRG in controlling dendritic arborization, which will distinguish between NRGs that function during the development of neuronal architectures from those that function in maintenance and stability of dendritic structures. Candidates that function in maintenance and stabilization of dendritic arbors, as opposed to those that are required throughout development, may be attractive candidates for therapeutic intervention.
